# Probing the Neural Circuitry Targets of Neurotoxicants *In Vivo* Through High Density Silicon Probe Brain Implants

**DOI:** 10.3389/ftox.2022.836427

**Published:** 2022-04-25

**Authors:** Marcia H. Ratner, David H. Farb

**Affiliations:** ^1^ Department of Pharmacology and Experimental Therapeutics, Boston University School of Medicine, Boston, MA, United States; ^2^ Center for Systems Neuroscience, Boston University, Boston, MA, United States

**Keywords:** *in vivo* electrophysiology, local field potential (LFP), place cell recordings, behavioral testing, neural network

## Abstract

Adverse effects of drugs on the human nervous system are rarely possible to anticipate based on preclinical neurotoxicity data, thus propagating the centuries long single most important obstacle to drug discovery and development for disorders of the nervous system. An emerging body of evidence indicates that *in vivo* electrophysiology using chronically implanted high-density electrodes (ciHDE) in freely moving animals is a rigorous method with enhanced potential for use in translational research. In particular, the structure and function of the hippocampal trisynaptic circuit (HTC) is conserved from rodents to primates, including *Homo sapiens*, suggesting that the effects of therapeutic agents and other potential neurologically active agents, whether beneficial or adverse, are likely to translate across species when interrogated using a conserved neural circuitry platform. This review explores science advances in the rapidly moving field of *in vivo* ciHDE in animal models of learning and memory. For this reason we focus on the HTC, where substantial research has investigated neural circuitry level responses and specific behaviors that reflect memory permitting a test of the ground truth validity of the findings. Examples of changes in neural network activity induced by endogenous neurotoxicants associated with neurodegenerative diseases, as well as exogenous therapeutics, drugs, and neurotoxicants are presented. Several illustrative examples of relevant findings that involve longer range neural circuitry outside of the HTC are discussed. Lastly, the limitations of *in vivo* ciHDE as applied to preclinical neurotoxicology are discussed with a view toward leveraging circuitry level actions to enhance our ability to project the specificity of *in vitro* target engagement with the desired psychopharmacological or neurological outcome. At the same time, the goal of reducing or eliminating significant neurotoxic adverse events in human is the desired endpoint. We believe that this approach will lead to enhanced discovery of high value neuroactive therapeutics that target neural circuitry domains as their primary mechanism of action, thus enhancing their ultimate contribution toward discovery of precision therapeutics.

## Introduction

The mapping of neurotoxicant actions at the neural circuitry level remains an under explored territory of preclinical neurotoxicology. As we strive to understand the effects of toxicants and therapeutics on the human nervous system the field of research has shifted away from testing in animal systems for various reasons. However, a broad view of neurotoxicology encompasses toxic side effects that present as adverse events, which continue to be a major obstacle in drug discovery (1) and often shows up in late-stage clinical trials or worse yet following post-marketing distribution. But can we leverage advances in the area of neural circuitry to understand the mechanisms by which neurotoxicants act and based upon this knowledge build a better platform for guiding discovery of therapeutics?

Preclinical neurotoxicology using animal models has historically relied on determining the changes in behavior and/or neuropathology in response to increasing doses of exposure, defining “biological markers of effect” that are then correlated with “biological markers of exposure,” such as blood levels of biomarkers, to establish drug safety profiles. This methodology has proven to be highly effective, yet a new opportunity has emerged in the form of *in vivo* brain implants bearing nanotechnology manufactured silicon probes for circuitry level electrophysiology.

All of the information acquired is valuable toward gaining an understanding of the biological basis of toxicology. Yet toxicology seeks to go beyond this goal and translate the results into actionable outcomes of importance to public health. In this respect, the sensitivity and specificity of behavioral tests alone is limited by the applicability of animal behavioral models and differences between animal performance on the specific behavioral tests used (Kulig et al., 1996; Vezér et al., 2005; Hã¥nell and Marklund, 2014). As a result, achieving sufficient statistical power to perform meaningful analyses of the variance in performance on behavioral tests often requires relatively large groups of rodents at great cost. When between subject designs are combined with these same behavioral tests, the inherent variability in animal performance requires even larger size groups (e.g., 12 or more animals per group) (Vezér et al., 2005). Behavioral studies also require multiple blinded investigators to score the raw data, contributing an unavoidable element of observer bias and additional variability (Haggerty, 1991; Vezér et al., 2005; Bohlen et al., 2014). For example, observer-scored assays are subject to unconscious scorer-bias the and require evidence of high inter-scorer reliability to ensure reproducibility (Haggerty, 1991; Bohlen et al., 2014). These concerns may be mitigated using automated objective measurements of animal behavior (Sturman et al., 2020).

Though the impact of rodent models on drug discovery for human diseases remains controversial (McGonigle and Ruggeri, 2014) the use of humanized transgenic animals has added to risk/benefit ratio estimates for novel therapeutics. For neurodegenerative disease research, interpretation of the results is further complicated by differences in subclinical prodromal neuronal responses to therapeutic candidates and how those effects translate to effective treatment outcomes. The need for improved preclinical models to assess adverse chemical effects on cognition is essential and has been noted and reviewed extensively (Sarter, 2006; McGonigle and Ruggeri, 2014; Mathiasen and Moser, 2018; Gulinello et al., 2019).

Neuropathological methods are relatively sensitive and biological markers for neurotoxicant effects can provide evidence of necrosis and apoptosis. Biomarkers for increased reactive oxygen and nitrogen species along with neuroinflammation can collectively establish drug neurotoxicity (Ali et al., 1992). Such studies typically require between subject designs because subjects must be euthanized to harvest brain tissue. This methodological limitation leads to the use of large groups of animals, stratified by dose, to achieve statistical significance between matched doses of neurotoxicants (Sakamoto et al., 2000).

Fluid-based biomarkers of neurotoxicity such as glial fibrillary acidic protein, myelin basic protein, microtubule-associated protein-2, Aβ and tau permit detection of neurotoxic effects without the need for euthanasia. However, while some of biomarkers correlate well with diseases such as Alzheimer’s, the correlation with neurotoxicant-induced behavioral changes requires further validation. Direct evidence that functional changes within the nervous tissue precedes overt behavioral effects needs validation (Roberts et al., 2015).

Functional neuroimaging provides evidence of changes in regional cerebral blood flow and magnetic resonance spectroscopy can detect metabolic markers of neuronal viability, such as *N*-acetyl aspartate. Although neuroimaging has several advantages over neuropathology, neither method monitors real-time network activity of specific neuronal cell types such as pyramidal cells and interneurons in a circuit. However, a combination of these techniques in conjunction with *in vivo* ciHDE can detect the unitary and synchronous actions within the brain parenchyma, including neurons, glial cells, and neurovascular units that are known to be vulnerable to neurotoxic effects of exogenous and/or endogenous toxins.

## Future Directions: The Role of Chronically Implanted High-Density Electrodes *In Vivo* Electrophysiology in Toxicology

**FIGURE 1:**
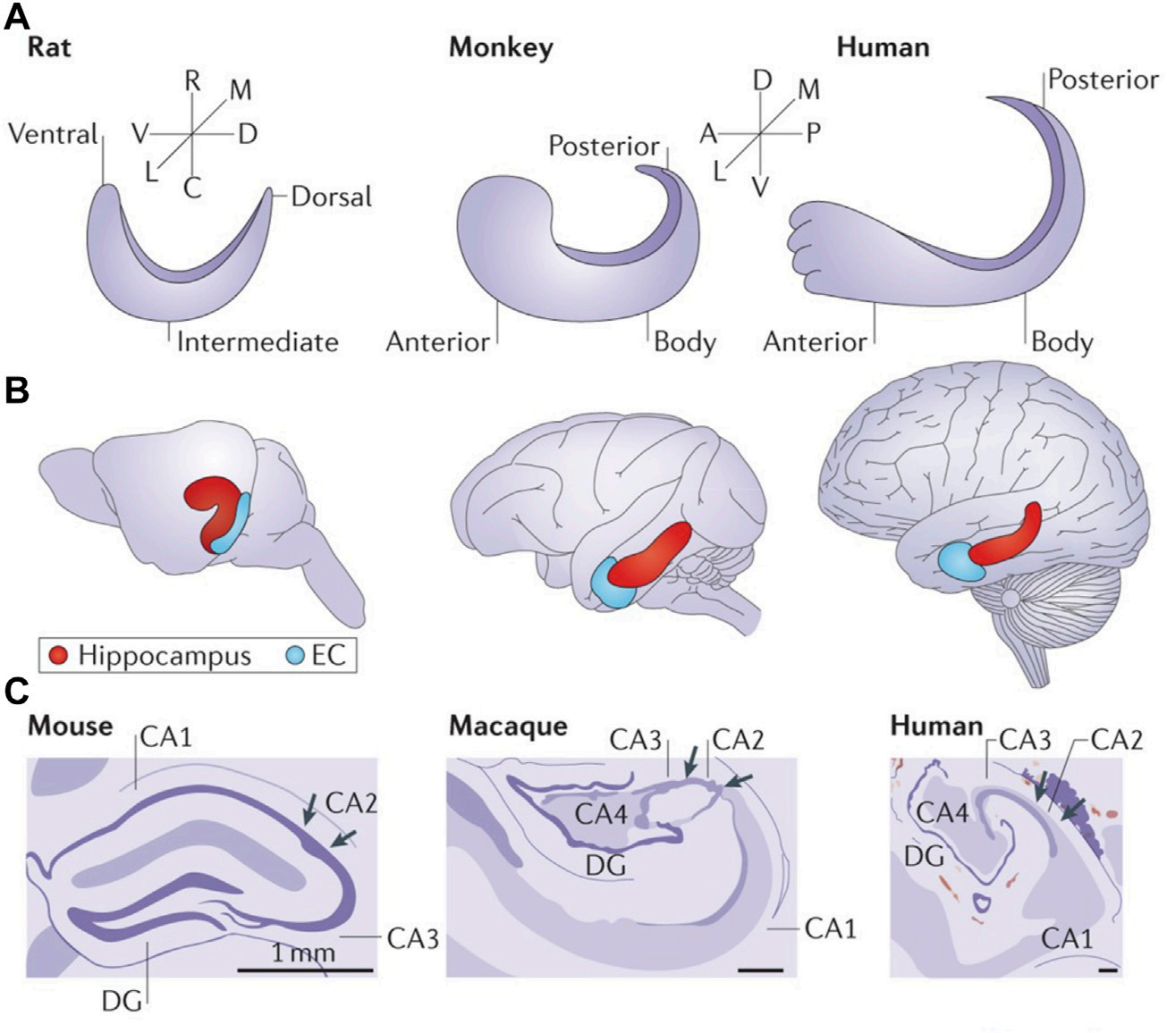
The shared functional organization of the hippocampus in rodents, non-human primates and humans contributes to the advantage of *in vivo* ciHDE recordings of neural activity from this brain region. **(A)** Orientation of the longitudinal axis of the hippocampus in rats, macaque monkeys and humans. The longitudinal axis is referred to as ventral/dorsal in rodents and as anterior/posterior in non-human primates and humans. **(B)** The long axis of the hippocampus is shown in red with the entorhinal cortex (EC) in blue. **(C)** Artists renderings of Nissl stained cross-sections of hippocampi from rodent (mouse), non-human primate (rhesus macaque monkey) and human reveal the conserved neuroanatomy. A, anterior; C, caudal; D, dorsal; DG, dentate gyrus; L, lateral; M, medial; P, posterior; R, rostral; V, ventral. Reproduced with permission (Strange et al., 2014).

Beyond our fascination with understanding simple systems for learning and memory, the observation that the structure and function of the hippocampal formation is highly conserved and plays a constitutive role in memory from rodents to non-human primates and human (Strange et al., 2014) (Figure 1) provides a basis for positing that what is learned from one species has relevance to the other**.** Moreover, the HTC is one of the most well-studied brain circuits because of its central role in learning and memory and its conservation across species.

The hippocampus is vulnerable to adverse effects of drugs and neurotoxicants (Tsunashima et al., 1998; Campbell et al., 2004; Bowyer and Ali, 2006; Bowyer et al., 2008; Ruden et al., 2021) such as impaired learning and memory during development that interferes with acquisition of language and academic skills. A particular challenge presents with aging adults that often use multiple medications. Off target effects in combination with compromised detoxification may adversely affect memory and it is unknown whether this might be exacerbated by or contribute to neurodegenerative diseases such as Alzheimer’s.

The foundation for using *in vivo* ciHDE electrophysiology in pharmacology and toxicology is based on its impressively reliable use in recording from diverse brain regions, from the neocortex and allocortex, to subcortical structures and cerebellum, among others. It all began with the pioneering work of O’Keefe and Dostrovsky (1971) who demonstrated that rat hippocampal pyramidal cell firing rates correlate with spatial location. The subsequent work of György Buzsáki et al. (1992) demonstrated a role for hippocampal sharp wave ripples (SPW-Rs) in the transfer of information between hippocampus and neocortex. Of course, many other distinguished leaders contributed to building this field and its underlying technology, including our collaborator Howard Howard Eichenbaum et al. (2016).

Here, we explore the idea that *in vivo* ciHDE has emerged as an objective, rigorous, and reproducible method for investigating neurotoxicity based upon the circuitry of memory. For example, SPW-Rs are observed in the CA3 and CA1 regions of the hippocampus within local field potentials (LFPs). These unique patterns of activity are composed of a negative “sharp-wave” associated with the synchronous discharge of CA3 pyramidal cells and high frequency (140–200 Hz) oscillations of CA1 pyramidal cells called “ripples.” SPW-Rs are believed to be packets of historical information conducting the consolidation and transfer of information from the hippocampus to the neocortex*.* When taken together, research advances have effectively bridged the gap between neural circuitry and spatial memory in rat and human (O'Keefe and Dostrovsky, 1971; Buzsáki et al., 1992; Axmacher et al., 2008; Schölvinck et al., 2010; Hargreaves et al., 2012; Fujita et al., 2014; Robitsek et al., 2015; Eichenbaum et al., 2016; Vaz et al., 2019; Ratner et al., 2021). Discovering how neural activity patterns are altered to yield memory dysfunction in age-related mild cognitive impairment (aMCI) and Alzheimer’s disease (AD) (Robitsek et al., 2015; Ratner et al., 2021) presents a major challenge and opportunity for translational research.

That said, it is important to recognize that the purpose and goal of using *in vivo* ciHDE as an “advanced applied science” differ from classical use in conjunction with trained behaviors to elucidate how neurons communicate related to their standard use combined with specific observational behavioral sequalae. Rather, our purpose is to interrogate specific neural circuits for potential neurotoxicity. The dose-dependent changes in neural network activity induced by acute and chronic administration of the compound(s) of interest can determine whether neural activity returns to baseline after the drug is cleared *via* excretion or has long lasting effects. These complementary approaches will almost certainly reveal unrecognized effects of drugs on neural network function and inform our understanding of memory itself.

This powerful objective method shows predictive and construct validity when recordings are made from conserved brain regions such as the hippocampal formation (Strange et al., 2014; Roberts et al., 2015; Robitsek et al., 2015; Ratner et al., 2021). *In vivo* ciHDE recordings can also be combined with behavioral models with high ethological validity such as open field exploration, novelty object recognition, and novel location exploration, wakeful resting and sleep (Robitsek et al., 2015; Ratner et al., 2021). The extrinsic validity of this method which yields similar results when performed with different rodent strains, age groups, and species is also high (Ratner et al., 2021). Furthermore, LFPs can be recorded from rodents, non-human primates, and humans (Axmacher et al., 2008; Eschenko et al., 2008; Schölvinck et al., 2010; Hargreaves et al., 2012; Fujita et al., 2014; Buzsáki, 2015; Robitsek et al., 2015; Eichenbaum et al., 2016; Trimper et al., 2017; Vaz et al., 2019; Ratner et al., 2021). An emerging body of literature from rodent and non-human primates demonstrates that data obtained with ciHDE is highly correlated with that obtained with functional imaging studies (Hargreaves et al., 2012; Vaz et al., 2019). Upon completion of ciHDE, hippocampal tissue from the implanted animals is harvested for documentation of electrode placement on the ipsilateral side and the contralateral hemibrain to be used for other purposes such neuropathological studies.

Studies in patients with drug-resistant epilepsy using subdural electrodes placed along the medial temporal lobe and other areas of the cortex have demonstrated that ripple oscillations play a role in episodic memory function in humans (Axmacher et al., 2008; Schölvinck et al., 2010) as well as rodents and non-human primates (Fujita et al., 2014; Robitsek et al., 2015; Eichenbaum et al., 2016; Ratner et al., 2021), demonstrating the potential of ciHDE. The findings from functional magnetic resonance spectroscopy studies of regional cerebral blood flow in aging humans with memory deficits are also consistent with ciHDE in aging rodents (Bakker et al., 2012; Robitsek et al., 2015; Bakker et al., 2015). Such observations indicate that such a functional circuitry level approach offers promise as part of our efforts to determine efficacy, potency, and most importantly safety of therapeutics.

## Key Concepts: Advantages of *In Vivo* Chronically Implanted High-Density Electrodes Electrophysiology

In this section we explore the key concepts of *in vivo* ciHDE, along with its strengths and weaknesses in preclinical neurotoxicology as compared with other electrophysiological methods. For single cell sharp electrode or patch clamp recordings, electrophysiology is used to measure drug-induced responses from cultured cells (*in vitro*) and in *ex vivo* brain slices (maintained *in vitro*). Using extracellular recordings, *in vivo* from chronically implanted brain regions. Each of these methods has unique advantages and disadvantages (Table 1). Single cell *in vitro* electrophysiology permits the measurement of ion currents across membranes of neurons, muscle fibers, and oocytes overexpressing single ion channels. *Ex vivo* slice electrophysiology permits the study of electrically active networks using microelectrodes embedded within the slice and/or individual neurons, permitting measurement of extracellular field potentials and intracellular fast ion currents. Long-term potentiation (LTP) and long-term depression (LTD) have been prominently used to suggest potential effects on memory *in vitro*. *In vivo* ciHDE uses surgically implanted indwelling microelectrode arrays to record extracellular action potentials and LFPs which combined with the behavior of the freely moving animal can be used to bridge from molecular target, through neural circuit to behavior.

**TABLE 1:** Advantages and disadvantages of different electrophysiological methods.

Preparation	Recording conditions	Advantages	Disadvantages
Single cells	*In vitro* with single recording electrode	Excellent time resolution of drug actions at specific receptors and receptor subtypes	No relationship to cytoarchitecture of synaptic circuits of an intact brain. No direct relationship to biologically relevant concentrations of systemically administered drugs or drug metabolites
Neuronal networks	*In vitro* with multi-electrode arrays	Spontaneous neuronal action potentials and oscillatory activity	Lack inputs and outputs that exist in the intact brain. Findings cannot be correlated with any real-time behaviors. No direct relationship to biologically relevant concentrations of systemically administered drugs or drug metabolites
Brain slice	*Ex vivo* with single electrode or multi-electrode arrays	Retention of local natural cytoarchitecture and synaptic circuits of the intact brain region (e.g., hippocampus)	Lack sensory inputs that exist in the intact brain. Findings cannot be correlated with any real-time behaviors. No direct relationship to biologically relevant concentrations of systemically administered drugs or drug metabolites
ciHDE	*In vivo* multi-electrode arrays, using freely moving animals	Retention of global *in vivo* cytoarchitecture and synaptic circuits of an intact brain. Measurement of fast single unit activity and slow LFPs within and across strata. Use of orally and systemically administered drugs and/or behavioral environments for within subject responses to systemically administered drugs at biologically relevant concentrations	Surgical implantation of indwelling electrodes can transiently disrupt the blood brain barrier locally, stimulate gliosis immediately around the recording shank, depending upon its size

The first three approaches utilize methods that reduce the use of animals in toxicology research. But each lacks intact *in vivo* connectivity between brain regions that underlies memory and cognition. Disconnection from *in vivo* systems ablates the constitutive relationships between brain and body, such as the hepatic and renal systems that underlie drug metabolism and bioavailability. Systems connectivity *in vivo* is essential to assess potential neurotoxicity and translate dose-dependent preclinical findings to precision therapeutics in human.

Advantages of *in vitro* single cell and *ex-vivo* slice electrophysiology include excellent time resolution of drug actions at specific receptors and receptor subtypes. In both instances recording electrode is inside one neuron at a time. Stimulating electrode may or may not be used. Drug application with pipet or “Puffer” is located near the perikaryon but can be located distally as well (Choi et al., 1977; Choi et al., 1981). This method is well-suited for investigating dose-response curves (Chan and Farb, 1985). Drugs can also be applied *via* iontophoresis or osmotic pressure. Focal stimulation with a stimulating electrode can be used for fine mapping of response to excitatory activity. Disadvantages of this method include non-existent relationship to cytoarchitecture of synaptic circuits between all innervated regions of an intact central and peripheral nervous system, and circulatory systems of a subject.

Neuronal networks grown on multi-electrode arrays are a cost effective solution to record spontaneous neuronal action potentials and oscillatory activity, but lack inputs and outputs essential to brain function. Isolated from the *in vivo* environment, the findings cannot be linked to real-time behaviors within subject though important correlations can be made.

*In vivo* ciHDE is the only electrophysiological method that retains the inputs and outputs as well as the cytoarchitecture and synaptic circuits of the intact brain with the added benefit of permitting real-time monitoring of observable behaviors during the measurement of neural network activity. In this way dose-dependent responses to systemically administered drugs including orally administered drugs can be determined in wild type and experimentally challenged animals (Buzsáki et al., 1986; Buzsáki et al., 2015; Robitsek et al., 2015; Ratner et al., 2021) (Table 1).

*In vivo* silicon probe ciHDE measures the extracellular changes in voltage (e.g., in millivolts) over time concurrently with action potentials from individual pyramidal cells and interneurons located near each electrode. The aggregate activity of extracellular voltage fluctuations from pyramidal cells and interneurons comprise the LFPs that oscillate slowly. *In vitro* electrophysiological observations can expand mechanistically upon *in vivo* observations (Buzsáki, 2015). Benzodiazepines and cholinesterase inhibitors, therapeutics with known mechanisms of action, can help identify the pharmacology of neural circuitry and to what extent neurotoxicity alters the pathology or reversibly modulates the circuitry. As in all electrophysiology and imaging experiments, the raw data can be posted in online repositories accelerating ongoing efforts that increase transparency and reproducibility.

### Action Potentials

**FIGURE 2:**
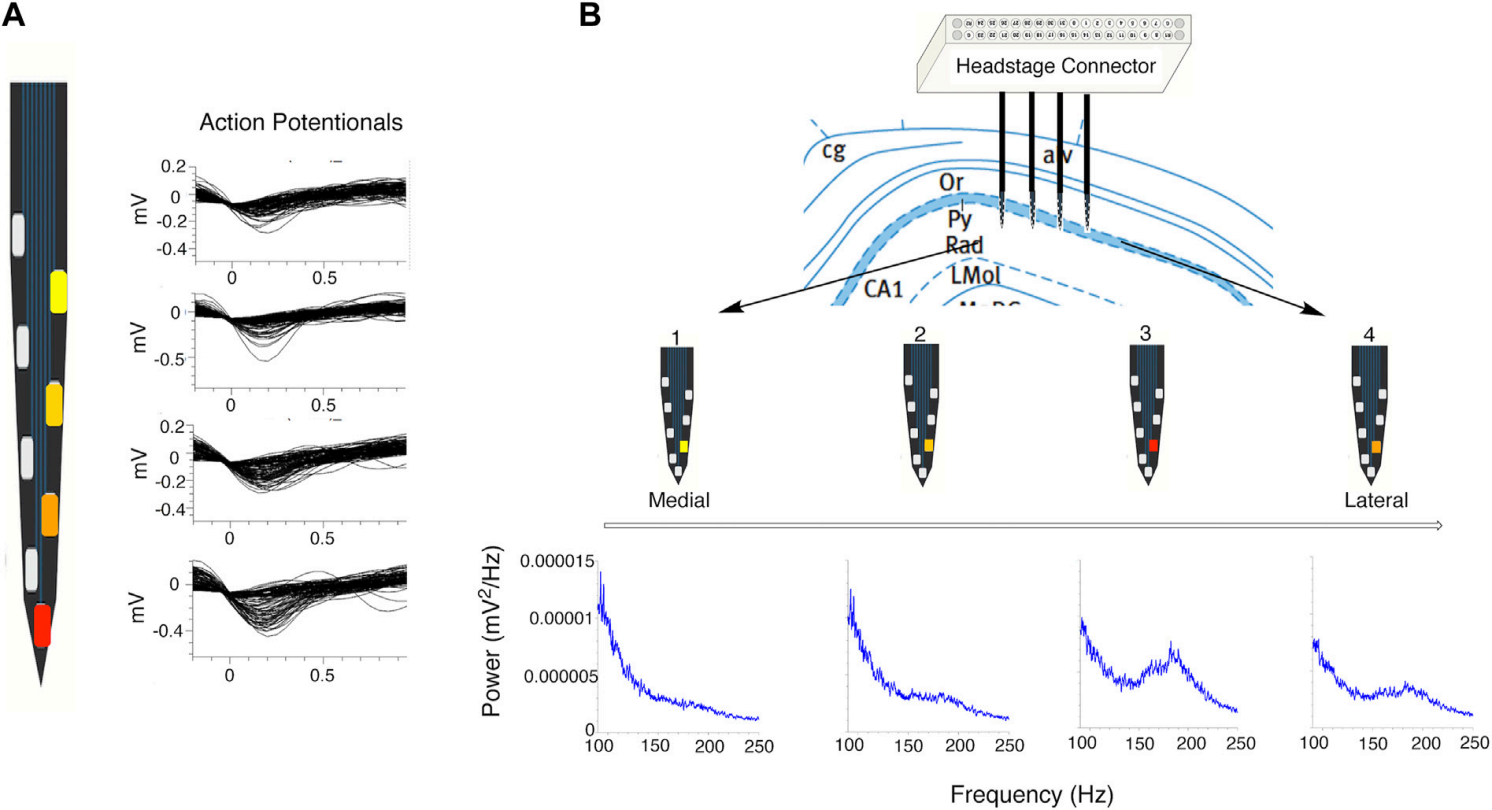
Effects of proximity to signal source on signal strength. **(A)** Close up of single shank of a silicon probe showing effect of proximity of a pyramidal cell to recording electrode (red electrode is closest to cell) on amplitude of representative action potential waveforms relative to background activity. **(B)** Representative location of silicon probe electrodes positioned in CA1 subregion. Power spectral density plots (shown in blue) demonstrate the effect of shank and electrode locations relative to the pyramidal cell layer on power in the ripple and epsilon bands (red electrode on shank 3 is the most well positioned within the CA1 pyramidal cell layer). Characteristic dip at 130 and 140 Hz between the epsilon and ripple bands is clearly seen on PSD plots recorded from the red and orange electrodes on shanks 4 and 3.

Extracellular recordings of action potentials detected with indwelling microelectrodes positioned near the cell body of a neuron are negative-going changes, reflecting a current sink created by the rapid influx of Na+ into the cellular unit. The measured amplitude of action potentials decreases with distance from the location of action potential generation, the axon hillock or other sites on the neuron, and orthograde propagation via the axon or dendrites. The neuronal perikaryon does not in general contribute to the action potential except as a passive conductor. The dependence of action potential amplitude due to distance from the current source/sink can be used to differentiate individual neurons from other nearby neurons via the cluster sorting process (Buzsáki et al., 2015) (see Figure 2A). The information about action potential amplitude and direction (positive vs. negative) can be combined with the known spacing between electrodes on silicon probes to determine the relative location of neurons and/or circuitry elements by virtue of current sources and sinks and to generate current density maps (Buzsáki et al., 1986). These current sources and sinks which reflect the architecture of the hippocampal formation are not anticipated to change if the recording electrodes are not moved when a within subject design is used for *in vivo* dose-response studies (Ratner et al., 2021).

**FIGURE 3:**
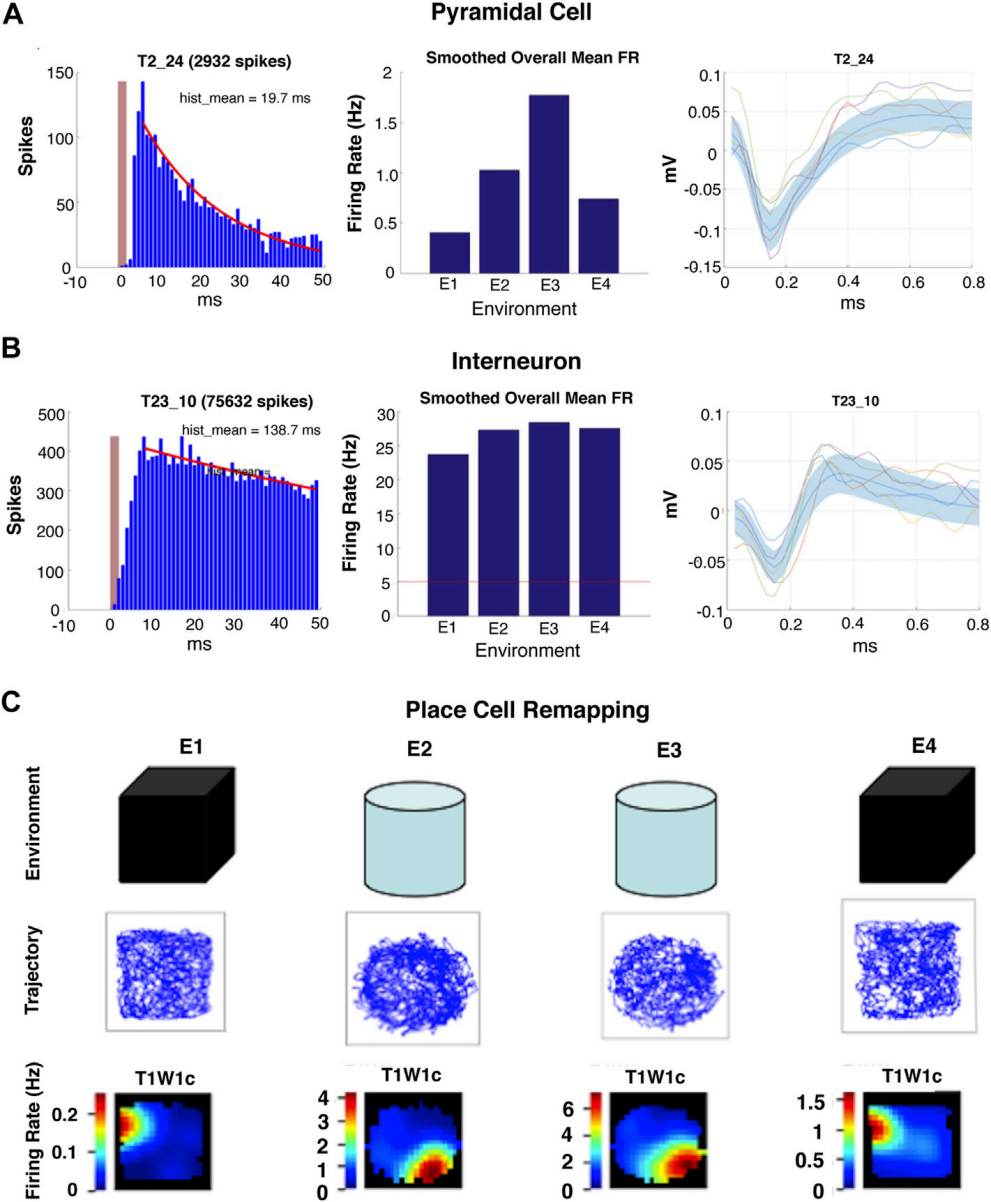
Examples of types of single cell activity recorded with *in vivo* ciHDE. **(A,B)** Auto-correlograms (left panel), mean firing rates histograms (center panel) and action potential waveforms (right panel) of pyramidal cells and interneurons. **(C)** Example of hippocampal pyramidal “place cell” remapping during serial exposure to a familiar (E1 and E4) and novel environment (E2 and E3).

Pyramidal cells can be distinguished from interneurons by several key features, including the: 1) mean firing rate (MFR), which is typically lower in pyramidal cells than interneurons; 2) waveform duration, which is typically longer in a pyramidal cell; and 3) presence of a characteristic burst of activity within the first 3–10 milliseconds after the refractory period as seen on an auto-correlogram (Fox and Ranck, 1975; Fox and Ranck, 1981; Skaggs et al., 1993; Csicsvari et al., 1998; Csicsvari J et al., 1999). (Figures 3A,B). Hippocampal pyramidal cells form stable firing patterns with specific “place fields” wherein these cells discharge at a higher relative rate in specific locations on initial and repeated exposure to the same environment (Robitsek et al., 2015; Ratner et al., 2021). These neurons change their firing rates (rate remapping), spatial locations (spatial remapping), or both (global remapping) of their respective place fields upon exposure to novelty in the environment. The characteristic remapping of place cells is based on the comparison of a preexisting map with that of a new or novel environment, and for this reason the environment specific changes in place cell firing patterns are considered to be a biological correlate of spatial memory or some would say all memory (Skaggs et al., 1993; Agnihotri et al., 2004; Cacucci et al., 2008; Robitsek et al., 2015; Ratner et al., 2021) (Figure 3C). Ground truth studies with juxtaposed intracellular recording electrodes demonstrate the validity of the observed differences in extracellular recordings of pyramidal cells and interneurons whether manual or auto-sorting methods are used to differentiate and classify the action potentials of individual cells (Harris et al., 2002).

### Local Field Potentials

In addition to the action potentials of individual neurons, the aggregate of all the ionic currents derived from activity of neurons, glial cells and cells of the microvasculature occurring in the vicinity of an in dwelling microelectrode are recorded as LFPs. These LFPs, also referred to as “slow waves,” reflect information flow within and between specific subregions and strata of the hippocampus (e.g., CA1 or CA3 pyramidal layer, stratum oriens, stratum radiatum) and reflect the source of circuitry activity. Such depth-specific signals can be used for functional localization of electrodes during real-time data acquisition (Buzsáki et al., 1986; Hargreaves et al., 2012; Buzsáki et al., 2015; Ratner et al., 2021) (Figure 2B).

Silicon probes provide key information necessary to evaluate drug-induced changes in signals emanating from different hippocampal strata (Buzsáki et al., 2015). Because the unique features of LFPs are relatively conserved across strains and species of rodents (mice vs. rats) and across humans and non-human primates this method displays a relatively high level of extrinsic validity (Hargreaves et al., 2012). In addition to detecting epileptiform activity (Schölvinck et al., 2010), there are several key frequency bands of LFPs that have been extensively investigated with in the hippocampus of rodents. Frequency bands of LFPs observed in the hippocampus of rodents include: 1) Delta (1–4 Hz) which is seen during sleep and under anesthesia; 2) theta (5–10 Hz) which is seen during periods of exploration and rearing increases in frequency with running speed (Buzsáki, 2002; Harris et al., 2002; Sheremet et al., 2019); 3) Beta (15–30 Hz) which is associated with olfactory function; 4) Gamma (30–130 Hz) which is correlated with theta and also increases in frequency with running speed (Csicsvari T et al., 1999; Colgin et al., 2009); and 5) Ripples (140–200 Hz) which occur when animals are immobile and during slow wave sleep (Hargreaves et al., 2012; Buzsáki, 2015; Ratner et al., 2021), displaying increased ripple amplitude in response to novelty (Csicsvari T et al., 1999; Csicsvari et al., 2000; Buzsáki, 2015). Ripples also occur during consummatory behaviors (Buzsáki, 2015). Sleep spindles (12–15 Hz oscillations), which originate in the thalamus, are visible in LFPs recorded from the neocortex during non-rapid eye movement (non-REM) sleep (Andrillon et al., 2011; Sullivan et al., 2014) (Table 2).

**TABLE 2:** Characteristic of LFPs recorded from the hippocampus of freely behaving rodents.

Frequency name	Range (Hz)	Associated behaviors
Delta	1–4	Sleep and anesthesia
Theta	5–10	Ambulation; increases with running speed
Sharp waves	5–15	Immobility and slow wave sleep
Sleep spindles	12–15	Non-REM sleep
Beta	15–30	Olfactory function
Low gamma	30–55	Ambulation; increases with running speed
High gamma	60–90	Ambulation; increases with running speed
Epsilon	90–130	Sleep and run
Ripples	140–200	Consummatory states, immobility and slow wave sleep

The frequency of activity in the theta band increases with running speed. The firing of pyramidal cells in the CA1 subregion shows a temporal relationship with theta activity referred as spike timing or phase coupling. When rodents are ambulatory the relationship between hippocampal theta and CA1 pyramidal cell firing shows a unidirectional advancement or “phase precession” as the animal moves into and out of the place field. This functional relationship is implicated in the temporal encoding of spatial and non-spatial information (Cacucci et al., 2008). The theta to delta power ratio is often used to functionally differentiate periods of movement from periods of immobility and sleep (Buzsáki et al., 1986; Ratner et al., 2021). The ordinal intercept of activity in the theta frequency band is sensitive to sedative hypnotics (e.g., benzodiazepines) (Wells et al., 2013). Theta oscillations show phase reversal of the LFP signal across hippocampal layers (Buzsáki et al., 1986). The amplitude of theta oscillations is greater in the hippocampal fissure than in the pyramidal cell layer of CA1 allowing for this functional variation to be used to predict electrode location in real-time during recording (Buzsáki et al., 1985).

Gamma oscillations are typically divided into three functional sub-frequencies: 1) low gamma (30–55 Hz); 2) high gamma (60–90 Hz), and 3) fast gamma, also known as epsilon (90–130 Hz) (Sullivan et al., 2011; Ahmed and Mehta, 2012; Buzsáki and Wang, 2012; Amemiya and Redish, 2018; Oliva et al., 2018). Power in the gamma and epsilon bands is modulated by the phase of spindle oscillations during sleep (Sullivan et al., 2014). There is a high correlation between theta and gamma power such that the activity in both frequency bands show an increase in mean frequency with running speed in four hippocampal strata (i.e., oriens, pyramidale, radiatum, and lacunosum moleculare) of the rat (Ahmed and Mehta, 2012; Amemiya and Redish, 2018).

**FIGURE 4:**
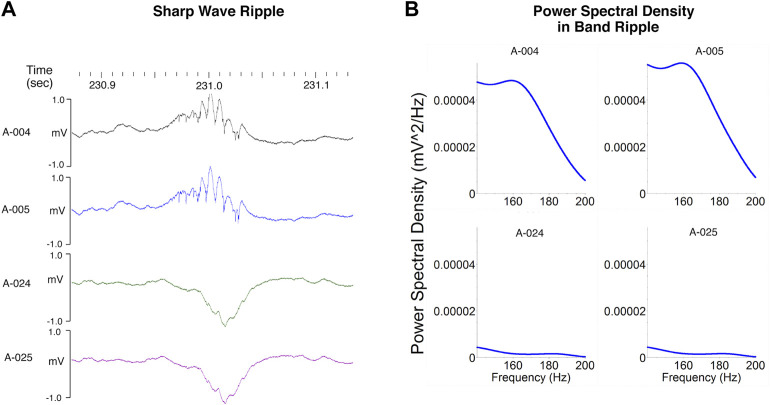
Sharp wave ripples. **(A)** Ripples (140–200 Hz) are seen in local field potentials recorded from electrodes well positioned within the CA1 pyramidal layer. These high frequency oscillations are associated with much slower (5–15 Hz) negative “sharp-waves” clearly visible on electrodes positioned below the layer where the apical dendrites of CA1 neurons are depolarized by the synchronous discharge of CA3 pyramidal cells. **(B)** The depth of the recording electrodes also influences power in the ripple band which is greater for those electrodes positioned in the CA1 pyramidal layer than for those positioned in stratum radiatum. The characteristic dip in power corresponding to the functional boundary between the epsilon and ripple bands is seen between 130 and 140 Hz. Modified from Ratner et al. (2021).

Ripples are high frequency oscillations (140–200 Hz) associated with much slower (5–15 Hz) negative “sharp-waves” triggered by the synchronous discharge of pyramidal cells in the CA3 subregion which in turn depolarizes of the apical dendrites of CA1 neurons (Hargreaves et al., 2012; Ratner et al., 2021; Csicsvari et al., 2000; Sullivan et al., 2011) (Figure 4). Ripples occur during periods of slow wave sleep and immobility when an animal is awake but resting quietly in its home cage or a familiar environment (Hargreaves et al., 2012; Ratner et al., 2021; Buzsáki, 2015). Although ripples and epsilon oscillations are quantitatively distinct activity patterns these two types of high frequency oscillations involve the same neural networks and share similar mechanisms (Sullivan et al., 2011). The biggest distinction between ripples and epsilon oscillations is that ripples are coupled to larger amplitude sharp waves (Buzsáki, 2015). A characteristic dip in frequency band power corresponding to the functional boundary between the epsilon and ripple bands is seen at 130 and 140 Hz (Buzsáki and Wang, 2012) (see Figures 2B, 4B). Ripples often occur in clusters 2 to 3 events (Hargreaves et al., 2012; Buzsáki, 2015). The synchronous discharge of at least 10% of CA3 pyramidal cells within a 100 ms time window is required to initiate a measurable ripple in CA1 subregion (Csicsvari T et al., 1999). Phasic inhibition mediated by parvalbumin expressing GABAergic interneurons locally modulate the amplitude of ripples in the hippocampal CA1 region (Gan et al., 2017). The peak amplitude of a ripple is associated with the highest amount of CA1 pyramidal cell activity (Csicsvari et al., 2000). Studies of non-human primates performing a memory task indicate that the amplitude of SPW-Rs is also associated with which trials are “remembered” and which are “forgotten” (Hussin et al., 2020). Ripples associated with trials that are not recalled have lower amplitudes. This observation is consistent with our findings demonstrating that the putative cognitive enhancer α5IA increases ripple amplitude (Ratner et al., 2021) and the observation that disruption of SPW-Rs impairs learning in rodents (Ego-Stengel and Wilson, 2009). Parvalbumin expressing interneurons are vulnerable to stressors and injury (Ruden et al., 2021) indicating that drugs and neurotoxicants that disrupt the functional integrity of these interneurons can be expected to interfere with SPW-R dependent memory processes.

## Current State of the Art: Changes in Neural Network Activity Associated With Exogenous and Endogenous Neuromodulators and Neurotoxins

As described above, *in vivo* ciHDE allows for investigations of the functional changes associated with systemically administered therapeutics and investigational drugs in combination with the neuropathology due to endogenous neurotoxic molecules such as Aβ (Ratner et al., 2021).

### Effects of Exogenous Modulators Affecting GABAergic Synaptic Transmission

We have previously shown that aging increases the mean and peak firing rates of hippocampal place cells while decreasing remapping of place fields with novelty in aMCI by using a within subject experimental design (Robitsek et al., 2015). In this study, we demonstrated the surprising finding that systemic co-administration of a single acute low dose of levetiracetam + valproic acid, both FDA approved anti-epileptic drugs, reversed the effects of aging in an aMCI rat model on a neural circuitry level although 2 weeks or more of drug treatment before having a demonstrable effect on behavior (Koh et al., 2010). Specifically, we used *in vivo* ciHDE with 96 electrode, 4-wire clusters called tetrodes implanted in CA3 and CA1 to show that LEV + VPA in a single acute dose restores spatial firing of place cells in aged rats. In addition, the low spatial information content (SIC), or the number of bits of place cell information per action potential, in aged rats was augmented to young adult SIC levels, without altering SIC or increasing place field quality in young unimpaired rats (Robitsek et al., 2015). These results are consistent with observations in human (Bakker et al., 2012; Bakker et al., 2015). While the mechanism of action for LEV + VPA remains unknown, LEV is believed to enhance synaptic vesicle protein 2 (SV2) (Lynch et al., 2004) modulated GABA release at presynaptic inhibitory terminals.

Again, based on the research from Michaela Gallagher’s findings, we were perplexed that positive (PAMs) but not negative allosteric modulators (NAMs) of alpha-5 subunit containing GABA-A receptors enhanced memory performance behaviorally in the aMCI rat model (Koh et al., 2010) as NAMs were shown to enhance memory performance in wild type mouse and rat (Rudolph and Möhler, 2006). We therefore used *in vivo* ciHDE to determine whether a decrease in alpha-5 subunit containing GABA-A receptor mediated tonic extrasynaptic inhibition and/or synaptic phasic inhibition is associated with an increase in CA1 ripple band power and SPW-R ripple amplitudes, such as might be expected for an enhancement of memory consolidation (Ratner et al., 2021). We found that α5IA (an investigational memory enhancer that was developed by Merck to preferentially inhibit alpha5 GABA-A receptors) potentiated the CA1 ripple band in wild type rats across multiple strains (Ratner et al., 2021). This provides compelling support for a key modulatory role for alpha5 GABA-A receptors in ripple dynamics, consistent with enhancement of ripples and memory consolidation. The results also indicate that the failure of an alpha-5 subunit containing GABA-A receptor NAM to enhance memory performance in the aMCI rat model (Koh et al., 2010) reflected the onset of the circuitry dysfunction with aging. These results await further experimentation to test the linkage between the positive modulation of ripple amplitude with memory enhancement measured behaviorally.

We also explored accelerating the time frame for neurotoxic effects of beta-amyloid by using a transgenic rat model for AD (the TgF344-AD rat) that overexpresses Abeta. We demonstrated that prodromal effects of beta-amyloid production occur in the HTC ripple band prior to measurable behavioral and pathological consequences. This early-stage biomarker for the onset of beta amyloid toxicity was revealed using α5IA as a probe drug. We found that it dramatically potentiates the amplitude of CA1 SPW-Rs in young adult WT F344 animals but that this effect is virtually eliminated in young adult TgF344-AD animals (Ratner et al., 2021). These observations indicate that administration of “probe drugs” with known memory enhancing effects can be used to “challenge” the neural circuitry and identify abnormalities induced by putative endogenous neurotoxicants that lead to or contribute to the onset of memory dysfunction in AD and other disorders. Probe drug can thus be used to predict the risk of adverse drug effects in these populations.

The slope and intercept of the linear plot of the relationship of the relationship between theta frequency and the animal’s running speed can be used to assess the effects of drugs and neurotoxicants. Systemic administration of the amnestic sedative benzodiazepine midazolam (1.5 mg/kg) decreases peak theta frequency by approximately 1 Hz and shifts the midpoint of the phase reversal towards the CA1 pyramidal cell layer (Balakrishnan and Pearce, 2014), although drug contributions to running speed could have been better controlled. The relationship between running speed and theta frequency at anxiolytic doses of benzodiazepines suggests that frequency is reduced but running speed is not significantly altered (Monaghan et al., 2017). Variation in the ordinate intercept component of the relationship between theta frequency in the CA1 hippocampal subregion and running speed in rats is reduced by administration of anxiolytics (Wells et al., 2013). Similar results are observed when recordings are made from the medial entorhinal cortex of adult male rats treated with 1.0 mg/kg of diazepam 30 min before testing (Monaghan et al., 2017). No effect of anxiolytics on slope, which is sensitive to environmental novelty, has been reported (Wells et al., 2013; Monaghan et al., 2017). By contrast, the anxiogenic drug FG7142, a non-selectively benzodiazepine positive GABA-A receptor modulator, has no significant effect on slope or intercept as a function of theta (Wells et al., 2013). *In vivo* ciHDE is anticipated to be useful in the assessment of dose-dependent effects of known and novel antidotes such as flumazenil aimed at reversing the acute neurological effects of GABAergic modulatory drug overdoses, such as from benzodiazepines.

In summary, it is well-known that GABA modulates brain function by regulating phasic and tonic inhibition via GABA-A and GABA-B receptors. Increases and decreases in GABA mediated inhibitory neurotransmission modulate ion flux, the frequency and amplitude of LFPs, and neuronal firing rates (Choi et al., 1977; Choi et al., 1981; Chan and Farb, 1985; Robitsek et al., 2015; Monaghan et al., 2017; Ratner et al., 2021). Monitoring changes in neural network activity measurable with ciHDE following systemic administration of GABAergic modulators depends in part on the basal level of neural circuitry function which can be interrogated using probe drugs selective for receptor subtypes preferentially mediating tonic inhibition vs. phasic inhibition in neuronal local circuits.

### Effects of Exogenous Cholinergic Modulators Affecting Synaptic Transmission

Cholinergic neurotransmission within the CNS is mediated by acetylcholine binding to neuronal nicotinic and muscarinic acetylcholine receptors. The muscarinic receptor antagonists atropine and scopolamine are both non-sedating amnestic agents (Newman et al., 2013; Balakrishnan and Pearce, 2014). Decreased cholinergic neurotransmission is also implicated in AD although this system has not thus far yielded efficacious treatments to slow neurodegeneration or decrease memory dysfunction (Stoiljkovic et al., 2018; Stoiljkovic et al., 2019). On a circuitry level, atropine (50 mg/kg shifts the phase reversal midpoint of theta away from the CA1 pyramidal cell layer without affecting peak theta frequency (Balakrishnan and Pearce, 2014). This response contrasts with that induced by midazolam, a positive allosteric modulator of phasic GABA-A receptors and amnestic, suggests that the network level changes induced by midazolam and atropine are induced by unique effects on theta activity. Peak gamma power in the medial entorhinal cortex occurred at later phases of theta following administration of scopolamine (0.5 mg/kg). In addition, the modulatory effect of running speed on theta was reduced by scopolamine (Newman et al., 2013). Systemic administration of donepezil, a reversible cholinesterase inhibitor, significantly increased theta phase-gamma amplitude coupling in the hippocampus during stimulation in a rat model of AD (Stoiljkovic et al., 2018). These findings demonstrate that *in vivo* ciHDE is well-suited for measuring the neuromodulatory effects of systemically administered drugs that alter cholinergic and GABAergic neurotransmission. This points toward a unique approach toward determining the neurotoxic effects of carbamate and organophosphate insecticides and chemical warfare agents.

### Effects of Exogenous Adrenergic, Serotonergic and Dopaminergic Modulators

Adrenergic, serotonergic and dopaminergic signal transduction processes mediated by G-protein coupled receptors are implicated in emotional and motor responses to many drugs and neurotoxicants. Systemic administration of the noradrenergic receptor agonist clonidine (30 μg/kg) suppresses the increase in CA1 subregion high gamma power normally seen in rats when they encounter choice points in a maze (Amemiya and Redish, 2018). LFP power in the striatum of mice treated with pseudoephedrine, or morphine reveal significant increases in power in the gamma range following treatment with morphine but not with pseudoephedrine as compared with saline baseline suggesting that pseudoephedrine dose have stimulatory effects of central nervous system function (Sa-Ih et al., 2020). Systemic administration of 8-OH-DPAT, a 5-HT7 receptor agonist and serotonin reuptake inhibitor, is associated with a decrease in the ordinate intercept of the theta frequency in the medial entorhinal cortex when running speed of the animal is equal 0 cm/s (Monaghan et al., 2017). No change in the slope which is sensitive to environmental novelty was observed (Wells et al., 2013). These findings are consistent with those seen in the hippocampus after systemic administration of other anxiolytic drugs known to produce memory deficits (Wells et al., 2013).

Tremor, bradykinesia, dyskinesia, and cognitive deficits are among the motor and non-motor symptoms seen in patients with disorders of dopaminergic neurotransmission such as Parkinson’s disease and neurotoxicant-induced parkinsonism (Zhu et al., 2011; Luca et al., 2021). *In vivo* electrophysiology can be used to elucidate the effects of altered dopaminergic neurotransmission on neural network activity. Dyskinesias are a profound and debilitating adverse effect of dopaminergic and antidopaminergic therapeutics seen in patients with Parkinson’s disease and schizophrenia. Cortical LFPs and neuronal spikes recorded from the motor cortex hemi-parkinsonian rats generated via unilateral injections of 6-hydroxydopamine (6-OHDA) into the medial forebrain bundle (MFB) reveal an increase in high gamma power (Dupre et al., 2016). Treatment with D1 agonist R(+)-SKF-81297 hydrobromide and the D2 agonist quinpirole also induced dyskinesia and an increase in high gamma activity like that induced by levodopa, indicating that this change in neural activity and the involuntary movements these dopaminergic modulators induce is neither D1 nor D2 receptor specific.

### Effects of Exogenous Glutamatergic Modulators

Glutamate is the principal excitatory neurotransmitter in the CNS and increased glutamatergic neurotransmission is implicated in excitotoxicity and induction of apoptosis (Lewerenz and Maher, 2015). Caixeta and others (Caixeta et al., 2013) showed that systemic doses of 25, 50 and 75 mg/kg of ketamine increased coupling between theta and high frequency oscillations (110–160 Hz) which include activity in the epsilon (90–130 Hz) and ripple bands (140–200 Hz) at all three doses, while theta-gamma coupling increased at the lowest dose but was disrupted at the highest dose. Acute systemic administration of the of the N-methyl-D-aspartate (NMDA) type glutamate receptors with the NMDA receptor non-competitive antagonist CPP [(±)-3-(2-carboxypiperazin-4-yl)propyl-1- phosphonic acid] is without effect on the size or quality of established CA1 place fields. By contrast, the long-term stability of placed fields formed on exposure to a novel environment was abolished by CPP administration (Kentros et al., 1998). The results demonstrate that glutamate and NMDA receptors are essential for the long-term stabilization of newly established place fields.

### Effects of Protein Synthesis Inhibitors

Protein synthesis is essential for all cells to function and survive and memory network function is acutely dependent on protein synthesis as well. Hippocampal pyramidal cells or place cells form stable firing patterns in specific locations as described above (Kentros et al., 1998; Agnihotri et al., 2004; Robitsek et al., 2015; Ratner et al., 2021). A potential linkage between the well-known dependence of LTP and memory on protein synthesis was demonstrated by the observation that administration of protein synthesis inhibitors interferes with long-term stability of CA1 place fields in mice (Agnihotri et al., 2004). The results demonstrate that protein synthesis as well as the proper balance between excitatory and inhibitory neurotransmission are essential to place cell stability acquisition (Kentros et al., 1998; Agnihotri et al., 2004).

### Effects of Exogenous Neurotoxicants

Exogenous neurotoxicants are substances that are not synthesized by the animal and, therefore, must be administered via various routes. Thiol-stabilized atomically precise gold nanoclusters (Au25(SG)18 nanoclusters) is used in cerebrovascular imaging, X-ray imaging, and cancer radiotherapy. The potential neurotoxicity of Au25 injection at high doses (200, 300, and 500 mg/kg) was investigated in mice using *in vivo* ciHDE. The acute increase in delta rhythm power in the CA1 subregion returns to baseline after all doses tested. No significant dose-dependent increases in the number of pyknotic neurons or glial fibrillary acidic protein (GFAP) was observed in the brain tissue of mice injected with Au25 at these concentrations indicating the observed reversible changes in neural activity were not associated with any permanent damage to the nervous system (Hao et al., 2020).

### Effects of Endogenous Neurotoxicants

Protein aggregates associated with some neurodegenerative diseases are examples of endogenous neurotoxicants. Well-known examples include amyloid plaques and neurofibrillary tangles in AD, and Lewy bodies in Parkinson’s disease. Amyloid precursor protein knock-in mice which begin to accumulate Aβ by 4 months of age and to show spatial memory impairments at 6 months show disrupted remapping of CA1 hippocampal place cells and reduced SIC per spike that correlates chronologically with spatial memory impairments (Cacucci et al., 2008). Analysis of LFPs from these mice reveal reduced high gamma and reduced gamma coupling between the medial entorhinal cortex and CA1 hippocampal subregion. Findings from *in vivo* ciHDE studies have also been correlated with biological fluid levels of Aβ to further improve relevance (Roberts et al., 2015; Ratner et al., 2021).

Rats overexpressing Aβ (TgF344-AD) show an age-dependent decrease in theta and decreased theta-gamma coupling (Stoiljkovic et al., 2019). Ratner et al. (2021) have shown that tonic disinhibition mediated by pharmacologically targeting α5 subtype GABA_A_ receptors increases peak ripple amplitude in wild type rats (see above). However, this response to probe drug is not seen in the TgF344-AD rat. How these changes in neural circuitry influence susceptibility to endogenous and exogenous neurotoxicants has not yet been elucidated.

Ciupek et al. (2015) showed that ripple band power and peak ripple amplitude are reduced in rTg4510 tau mice. The adverse effects of tauopathy on ripples also increases with age in these mice. Place cells of young tau mice had similar spatial information content per action potential (SIC) when engaged in familiar trajectories as WT mice, but place field stability of Tau mice was significantly lower. The authors noted that the adverse effects of tauopathy on ripples precedes the onset of memory deficits as well as alterations in place field stability. This suggests that ripples, which are implicated in memory consolidation, may be more sensitive to bioaccumulation of neurotoxic substances such as tau than behavioral performance or place cell activity. In future research, findings from *in vivo* electrophysiological studies should also be correlated with biological fluid levels of tau (Roberts et al., 2015).

Mice in which human α-synuclein is overexpressed show robust motor dysfunction in the absence of significant nigrostriatal dopamine degeneration. Lobb et al. (2013) used *in vivo* ciHDE to simultaneously record LFPs and single neuron activity in the substantia nigra pars reticulata (SNpr), as well as LFPs in the primary motor cortex in anesthetized head fixed mice overexpressing α-synuclein. Analysis of LFPs recorded in the SNpr, ventromedial thalamic nucleus, and primary motor cortex during slow wave activity and in pinch-induced desynchronized states revealed a small decrease in beta oscillations but no change in synchronized burst firing of nigral neurons observed in toxin-based models of parkinsonism indicating the pathophysiology underlying motor dysfunction in mice overexpressing α-synuclein may be different from that seen in dopamine-depletion models of parkinsonism. Mice that over-express human mutant α-synuclein (A30P) have normal SPW-Rs (Stylianou et al., 2020). These findings demonstrate the potential for *in vivo* ciHDE to objectively measure biomarkers of hippocampal function and to compare the effects of endogenous and exogenous neurotoxicants on the hippocampus and other vulnerable brain regions of interest.

Mild traumatic brain injury in mice significantly reduced the frequencies of ripples in CA1 and reduced CA1 ripple evoked responses in the medial prefrontal cortex (mPFC) while increasing theta-gamma phase amplitude coupling within the mPFC (Liu et al., 2017). Increased coupling of theta-beta oscillations within primary somatosensory and visual cortical areas was also observed in this model which was previously shown to produce diffuse axonal injury (Heldt et al., 2014).

## Limitations of *In Vivo* Chronically Implanted High-Density Electrodes Electrophysiology

As with any emerging high technology the successful use requires methods and analyses rife with pitfalls and limitations. First, noise due to electrical interference must be controlled. In addition to turning off non-essential electrical equipment, use of a Faraday cage and ensuring proper grounding during recording, a notch filter can be used after data acquisition to remove 60 cycle electrical noise from the signal. It should be noted that such an effect has not been demonstrated as a unique pitfall to *in vivo* ciHDE as it is a technical issue common to all electrophysiology. This illustrates, however, the fact that electrophysiology remains a highly specialized discipline even though dramatic advances in automation have occurred since our original development of automated high throughput electrophysiology (HTEP) using two-sharp electrode recordings from single cells in *ex vivo* culture (Farb, 1993; Yaghoubi et al., 1998; Gibbs et al., 1999; Farb et al., 2003; Farb et al., 2004). Second, surgical implantation of microelectrodes can disrupt the local integrity of the blood-brain barrier (BBB), though small in size can potentially allow limited focal entry of drugs and neurotoxicants to gain access to the CNS. Future research would benefit from an investigation into this possibility to affirm or refute it. Although a minor concern and notably limited in scope to the small regions surrounding the electrodes, this concern would apply to effects detected by compounds that are known not to penetrate the BBB. Third, implantation of chronic indwelling microelectrode arrays also causes local reactive gliosis which can alter signals. However, recent technological advances in scalable mesh probes suggest this limitation can be overcome even in rodent models (Fu et al., 2017). Fourth, when *in vivo* ciHDE is combined with demanding tasks such as maze learning, differences in animal performance can limit the value of data acquired. This limitation may be overcome by using additional animals and with less demanding models such as recording neural activity from animals while freely foraging for food in an open field or while immobile and resting in the home cage familiar environment (Ratner et al., 2021). Lastly, while interspecies differences in hippocampal function are unlikely to be a limitation, neurotoxicant induced dysfunction of the frontal lobes while performing complex executive functions may be limited by uncertainty of the underlying wiring.

Unlike basic behavioral equipment which are relatively inexpensive and technically simple to implement, *in vivo* ciHDE is technically difficult. In addition to experience working with animal behavioral assays and drug administration, this approach also requires the investigator to have expertise in various specialized methods. The ability to successfully perform delicate stereotaxic brain surgeries is essential. Experience with the collection, processing and analysis of electrophysiological data is also required. This type of expertise requires hands-on training which can be a significant barrier to entry. Data processing and analysis is increasingly facilitated by standardized commercially available software programs that improve reproducibility. MATLAB users have the option of adding signal processing apps and shared codes available on GITHUB. The availability of training videos published in open access online video journals by laboratories with extensive experience in this area has also made this method less difficult to adopt (Vandecasteele et al., 2012). Recent advances in robotic assisted stereotaxic surgery can be employed to reduce human error and improve data quality and the reproducibility of results across laboratories.

Cost is another barrier to entry. However, the development of open-source data acquisition equipment and analysis software currently available through open-ephys.org has fundamentally transformed the field and made this approach accessible to more laboratories. Adoption of automated signal detection methods is expected to improve the reproducibility of data acquired with *in vivo* ciHDE while also allowing for a greater number of applied researchers to successfully use this method in their investigations (Uygun et al., 2019). The cost of consumables such as prefabricated silicon microelectrode arrays remains a barrier to entry, but the cost of the small parts and fine nichrome wire necessary to construct a suitable custom microelectrode array is considerably lower (Kloosterman et al., 2009; Robitsek et al., 2015; França et al., 2020; Ratner et al., 2021).

Lastly, while interspecies differences in hippocampal function are unlikely to be a limitation to the translational value of *in vivo* ciHDE with respect to assessment of drug and neurotoxicant induced effects on memory function, the translational value of studies using ciHDE to assess for subtle drug induced changes in neural activity in the frontal lobes implicated in complex executive functions in humans remains to be determined.

## Highlights and Conclusion

The results described establish *in vivo* ciHDE as a rigorous method with great potential for reducing and refining the use of vertebrate animals in neurotoxicology research, particularly with respect to assessing adverse effects on learning and memory and many other applications. The existing data also demonstrate the high predictive, construct, and extrinsic validity of *in vivo* ciHDE in neurotoxicology. Although the rodent hippocampus and primate hippocampus differ in terms of their embryonic development, a cross-species comparison of anatomical connectivity provides evidence that the primate hippocampal long axis may be homologous to that of the rat (Strange et al., 2014) (see Figure 1). *In vivo* ciHDE appears to be uniquely well-suited for determining the adverse effects associated with multiple drug combinations (i.e., polypharmacy) which can impair learning and memory function in vulnerable aging populations (Assari et al., 2020). *In vivo* ciHDE also allows for an improved understanding of how systemically administered drugs differentially modulate neural network activity in healthy subjects and those with neurodegenerative diseases (Robitsek et al., 2015; Ratner et al., 2021).

LFPs vary with electrode location and specific behaviors but when the electrode location and behavior are controlled by recording from the same animals under the same experimental conditions before and after treatment (within subject), conclusions about dose-dependent drug effects on the frequency and amplitude of the LFP can be made reliably. For example, we have shown that recordings of SPW-Rs made in the same animals, on the same day in the same environment following treatment with vehicle or probe drug yield dose-response data that are reproducible across strains of rats (Ratner et al., 2021). This approach, in which *in vivo* ciHDE recordings are made under strictly controlled behavioral conditions with silicon probes, also enables the application of current density analysis of LFPs before and after probe drug or neurotoxicant administration. We have had similar success recording place cell activity before and after probe drug administration (Robitsek et al., 2015; Ratner et al., 2021). We also demonstrated that the effects of a systemically administered probe drug on neural network activity in specific brain regions of heathy control subjects does not reliably predict the neural responses of these same conserved brain regions in subjects with aMCI and neurodegenerative diseases such as AD (Robitsek et al., 2015; Ratner et al., 2021). These observations indicate this method can be used to screen previously identified potential therapeutics for unintended neurotoxicity in vulnerable populations. Based on our experience, investigating dose-response effects for novel therapeutucs and for repurposing studies are readily feasible (Robitsek et al., 2015; Ratner et al., 2021). Long-term recordings in humans with Deep Brain Stimulators indicate that LFPs can be recorded many months even years after implantation although signal amplitude may decrease with time (Abosch et al., 2012).

Based on the data presented here, we expect *in vivo* ciHDE to be useful for the following applications: 1) Measuring acute dose-dependent responses of neural circuitry to systemically administered investigational drugs and potential neurotoxicants; 2) Drug repurposing studies to determine dose-response effects and assess for unanticipated risks for neurotoxicity; 3) Identification of the neural network activity changes induced by specific classes of compounds (e.g., negative modulators of a5 type GABA_A_ receptors) which may have positive effects that outweigh risks in one patient population while not producing benefits that outweigh risks in others; 4) Identification of and monitoring of residual changes in neural network activity following cessation of exposure to investigational drugs and potential neurotoxicants.

In conclusion, *in vivo* ciHDE studies of neural activity reflect the functional integrity of highly conserved brain regions such as the HTC (see Figure 1). The ability to use silicon probes with known spacing between electrodes to differentiate real-time responses of current sources and sinks to systemically administered drugs is a unique strength of this methodology (Buzsáki et al., 2015). Neuropixel silicon probes which enable recording neural activity data from over 200 well-isolated neurons in the human brain provide an unprecedented opportunity for comparing human data to observations in preclinical models (Paulk et al., 2022). This approach has the potential to fundamentally transform the field of neurotoxicology and to advance our understanding of neurotoxic chemical effects beyond that attainable with electroencephalograpy (Freeborn et al., 2015). Despite the significant limitations of ciHDE, the high extrinsic and construct validity of *in vivo* electrophysiological recordings made from brain regions shared across species is expected to provide objective, rigorous, and reproducible results. We anticipate that the neural activity data acquired with *in vivo* ciHDE will be deposited in online neural data repositories for subsequent computational analysis using a neurotoxic database that we are developing to identify specific chemicals with actions on specific neural circuits, thereby further reducing the use of animals in preclinical neurotoxicology research, minimizing the need for replication of prior studies, and accelerating discovery in neurotoxicology. Looking ahead, we envision a future wherein cost-effective methods of *in vivo* ciHDE are combined with real-time functional and morphometric neuroimaging techniques in rodents and non-human primates to provide additional information about the location of recording electrodes and changes in regional cerebral blood flow to even more effectively demonstrate neurotoxic effects in these models while at the same time facilitating translation to humans (Pan et al., 2010; Jaime et al., 2018).

## Next Steps Toward a Better Understanding of Neurotoxicant Mechanisms of Action

Our stated goal in pharmacology and toxicology is to discover and develop safe and effective therapeutic agents with an emphasis on achieving treatment with little or no adverse effects but we lack a roadmap other than repeating the same kinds of tests and hoping to extrapolate the risk of engaging adverse events. To accomplish this, behavioral screens such as the Functional Observational Battery have historically been administered to noninvasively evaluate the onset, progression, and reversibility of gross changes in the behavior of rodents exposed to neurotoxic chemicals (Haggerty, 1991; Kulig et al., 1996; Vezér et al., 2005; Sarter, 2006; Bohlen et al., 2014; McGonigle and Ruggeri, 2014; Roberts et al., 2015; Mathiasen and Moser, 2018; Gulinello et al., 2019). The Functional Observational Battery is administered for evaluation of dose-dependent functional changes in the same animal over time, and the results are correlated with neuropathological findings to conclude the study.

The core preclinical measurements of motor function, coordination, sensory/motor reflexes and body temperature included in the Functional Observational Battery are reliable and reproducible. However, because the Functional Observational Battery is designed to detect gross functional changes, such as motor coordination, additional behavioral tests are necessary to evaluate the potential of a chemical and/or its metabolites and mixtures to impair cognitive function. The prevalence of neurological and psychiatric side effects seen with many if not all current medications used to treat neurological and psychiatric disorders bolster the need for testing higher order cognitive processes such as learning and memory in second-tier rodent neurotoxicity screens (Mathiasen and Moser, 2018; Gulinello et al., 2019). Behavioral tests currently used for this purpose include the novel object recognition, location novelty recognition, Morris water maze, Barnes maze, radial-arm maze, T-maze, Y-maze, and fear conditioning among others.

The same animal models that focus on performance in trained responding based behavioral paradigms are relied upon for the regulation of pesticides, metals, solvents, and gases encountered in the workplace and environment by the Occupational Safety and Health Administration (OSHA) and the Environmental Protection Agency (EPA) respectively. Reforms to the Toxic Substances Control Act call for new models and methods aimed at reducing and refining the use of vertebrate animals in preclinical neurotoxicology testing (United States Code of Federal Regulations, 2016). We posit that addition of an *in vivo* neural circuitry-based approach would add significance to existing methodologies enhancing the ultimate value of toxicological research to predicting risks of adverse events and/or injury to the human nervous system by chemical substances and their mixtures.
